# Alcoholic Liver Disease Is Associated with Elevated Plasma Levels of Novel Advanced Glycation End-Products: A Preliminary Study

**DOI:** 10.3390/nu14245266

**Published:** 2022-12-10

**Authors:** Kamil Litwinowicz, Ewa Waszczuk, Aleksandra Kuzan, Agnieszka Bronowicka-Szydełko, Kinga Gostomska-Pampuch, Piotr Naporowski, Andrzej Gamian

**Affiliations:** 1 Department of Biochemistry and Immunochemistry, Wroclaw Medical University, 50-368 Wroclaw, Poland; 2 Department of Gastroenterology and Hepatology, Wroclaw Medical University, 50-566 Wroclaw, Poland; 3 Hirszfeld Institute of Immunology and Experimental Therapy, Polish Academy of Sciences, 53-114 Wroclaw, Poland

**Keywords:** advanced glycation end-products, liver disease, alcohol

## Abstract

Elucidating the biochemical mechanisms associated with the progression of alcoholic liver disease (ALD) to more advanced stages such as alcoholic hepatitis (AH) remains an important clinical and scientific challenge. Several hypotheses point to the involvement of advanced glycation end-products (AGEs) in alcohol-associated liver injuries. Recently, we determined the structure of a synthetic, melibiose-derived AGE (MAGE), which was an analog of the novel AGE subgroup AGE10. The primary objective of our study was to determine whether AGE10 was associated with alcoholic hepatitis. The secondary objective was to provide a diagnostic accuracy of AGE10 in AH. To achieve this objective, we examined the plasma levels of AGE10 in 65 healthy individuals and 65 patients with AH. The AGE10 level was measured using a competitive ELISA. Our study confirmed that patients with AH had significantly higher plasma concentrations of AGE10 compared with healthy controls (184.5 ± 71.1 μg/mL and 123.5 ± 44.9 μg/mL, respectively; *p* < 0.001). In addition, AGE10 showed an acceptable performance as a diagnostic marker of AH, with an AUC of 0.78. In conclusion, AH was associated with elevated levels of novel advanced glycation end-product AGE10.

## 1. Introduction

The excessive use of alcohol is one of the most important contributors to global mortality. With 3 million deaths attributable to alcohol abuse, it accounted for 5.3% of all deaths in 2016. The effect of alcohol overconsumption on mortality is higher than HIV/AIDS, diabetes, or hypertension [1]. According to the World Health Organization, in 2016, approximately 132.6 million disability-adjusted life years were lost to the consequences of alcohol abuse [1]. The social and economic consequences of alcohol use disorder (AUD) are further exacerbated by the age structure of the deaths attributed to alcohol; deaths and disability from alcohol occur in relatively young people, with alcohol abuse being responsible for 13.5% of all deaths in people aged 20–39 years [1]. One of the leading causes of mortality in AUD is alcoholic liver disease (ALD) and its consequences. The most advanced form of ALD—alcoholic cirrhosis—accounts for 47.9% of all liver cirrhosis deaths [2]. Of all the chronic heavy drinkers, almost 100% will develop an alcoholic fatty liver. However, in this group, only 10 to 35% will progress to alcoholic steatohepatitis (ASH), and 8 to 20% will end up with cirrhosis [3]. The set of clinical features associated with ASH is called alcoholic hepatitis (AH). Elucidating the pathophysiological events responsible for the development of ASH and cirrhosis is of great clinical significance. The early detection of patients at a greater risk of progression to ASH would allow the development of more focused, individualized treatment approaches.

The diagnosis and accurate staging of ALD remain a challenge. A liver biopsy combined with a documented excessive consumption of alcohol remains the diagnostic gold standard for ALD. However, it is associated with a 2% risk of severe complications [4] and, as a consequence, the European Association for the Study of the Liver (EASL) guidelines for the management of ALD do not recommend its routine use [5]. For this reason, non-invasive tests of the liver function,—i.e., gamma-glutamyl transpeptidase (GGTP), alanine aminotransferase (ALT), and aspartate aminotransferase (AST)—remain the mainstay of an ALD diagnosis.

The invasive nature of a liver biopsy has prompted the development of novel, non-invasive tests for the accurate staging of ALD. Several biochemical parameters of liver fibrosis associated with ALD have been proposed. Currently, the individual biochemical marker with the highest diagnostic accuracy is hyaluronic acid [6]. However, biochemical panels combining multiple individual markers (such as an enhanced liver fibrosis test [7] or PGAA [8]) provide an even better diagnostic performance. In the case of alcoholic steatohepatitis, the spectrum of currently established biomarkers is much more limited, with the M30 and M65 epitopes of cytokeratin-18 (CK18) having the widest support in the literature [9]. Despite these early advances, it is worth highlighting that current guidelines do not recommend their use in clinical practice.

The pathophysiology of ALD is complex and involves multiple partially overlapping molecular pathways. The major pathophysiological foundations of ALD include the generation of reactive oxygen species (ROS) [3], microbiome alterations [10], and the accumulation of advanced glycation end-products (AGEs) [11]. AGEs, also called glycotoxins, are a group of long-lived, structurally diverse compounds. They are formed through several complex networks of biochemical reactions. The most important pathway begins with a reaction, which occurs between the reducing sugars and free amino groups of a protein, resulting in the generation of an unstable Schiff base. Subsequent rearrangements of the Schiff base lead to the formation of a stable Amadori product, which undergoes further oxidation, glycation, and cross-linking—generally called Maillard reactions—resulting in AGEs [12]. Both the Schiff base and Amadori product can further increase the rate of AGE generation by entering the Namiki and Wolff pathways, respectively, which produce dicarbonyls (such as methylglyoxal) [13]. Dicarbonyls are a group of highly reactive molecules, with up to 20,000 times higher glycating activity compared with glucose [14]. Apart from the Maillard reaction, dicarbonyls can also be generated through glycolysis, ketone body metabolism, lipid peroxidation, and the polyol pathway [15]. Although dicarbonyls are generally found in low concentrations in physiological conditions, due to their extreme glycating activity they significantly contribute to the formation of AGEs [14]. Their influence on the AGE pool can be further exacerbated in the pathological state; for example, hyperglycemia shifts glucose towards the polyol pathway, which leads to a significant increase in the generation of dicarbonyls [16].

The accumulation of AGEs has been hypothesized to play a role in a diverse range of diseases, including non-infectious liver disease [17], thyroid gland pathologies [18], diabetes [15], or even the development of psychotic symptoms [19]. Although the exact mechanism is still not fully elucidated, the involvement of glycotoxins in ALD is well-documented in the literature [17]. The relationship between AGEs and the development of liver disease appears to be bidirectional. On one side, the current leading hypothesis of AGE involvement in ALD (the so-called toxic AGE theory) postulates that interactions between acetaldehyde- or glyceraldehyde-derived AGEs and their receptor (RAGE) lead to the activation of molecular pathways, resulting in the release of proinflammatory cytokines and an increased generation of reactive oxygen species, which causes liver injuries [11]. On the other side, the liver is responsible for the removal of AGEs [20]; hence, a liver injury might lead to a further accumulation of glycotoxins.

To date, the molecular structure has been characterized only for a relatively small fraction of all AGEs (notable examples include carboxymethyl-lysine (CML), carboxyethyl-lysine (CEL), imidazolone, and pentosidine). Recently, the structure of a synthetic, melibiose-derived AGE (MAGE) was determined, which was an analog of a novel compound. As shown by the lack of cross-reactivity of anti-MAGE antibodies with other known subtypes of AGEs [21] as well as no significant correlation between MAGEs and fluorescent AGEs [18], this newly discovered subtype did not belong to any known group of AGEs; thus, it was called AGE10 [22]. Several interesting properties of MAGEs have already been confirmed. AGE10-modified proteins have been shown to be present in both diabetic patients and healthy controls, with different patterns of protein modification depending on the disease status; in diabetic patients, albumin and IgG were mainly involved whereas in healthy patients, it was mostly IgG and IgA [21]. The AGE10 concentration is significantly correlated with multiple risk factors for cardiovascular diseases such as hypertension and lower levels of HDL [23]. Serum from diabetic patients has been shown to contain MAGE-specific auto-antibodies [24]. In addition, synthetic MAGEs exhibited a significant genotoxicity against human peripheral blood lymphocytes and on multiple human cell lines [25]. In this study, we attempted to elucidate whether this novel subtype of AGE was associated with alcoholic hepatitis. As the early detection of AH is crucial for preventing irreversible damage to the liver, our secondary objective was to provide the diagnostic accuracy of AGE10 in ALD.

## 2. Methods

### 2.1. Participants

We included 65 patients with alcoholic hepatitis recruited at the Jan Mikulicz-Radecki University Teaching Hospital in Wroclaw, Poland. The inclusion criteria were an excessive intake of alcohol, a clinical diagnosis of alcoholic steatohepatitis (i.e., sudden onset or progression of jaundice, or increased levels of ALT or AST, with ALT and AST under 400 IU/l and an AST/ALT ratio over 1.5), and a lack of serious non-alcoholic-related comorbidities (such as HBV and HCV, renal dysfunction, hepatocellular carcinomas, sepsis, or multiorgan failure). An excessive intake of alcohol was defined as a daily consumption of more than 3 standard drinks for men and 2 for women (a standard drink corresponded with roughly 14 g of pure alcohol). A total of 65 healthy controls (HC) were recruited from the Tadeusz Dorobisz Regional Center for Blood Donation and Haemotherapy. The inclusion criteria for the controls were a lack of a significant medical history and the standard criteria for blood donation. All study participants gave their written consent; the study followed the guidelines of the Helsinki Declaration and was approved by the Ethics Committee of Wroclaw Medical University (numbers KN-713/2020, KB-187/2019).

### 2.2. Sample Handling

Whole blood samples were drawn into a vacuum blood collection tube containing EDTA. The collected samples were centrifuged. The supernatant was then aliquoted, frozen, and stored at −80 °C until the analysis.

### 2.3. Determination of AGE10 Content in the EDTA Plasma

The AGE10 content was determined using a competitive enzyme-linked immunosorbent assay (ELISA). First, 96-well MaxiSorp plates (Nunc^®^, Sigma-Aldrich, Darmstadt, Germany) were coated with synthetic high molecular mass MAGEs (HMW-MAGEs) for 5 h at room temperature. The plates were washed three times using phosphate-buffered saline (PBS) with 0.05% Tween-20 (PBST). The plates were then blocked overnight at 4 °C with 10% skimmed milk. The plasma samples were thawed at room temperature and 50 μL of each sample was taken into an Eppendorf safe-lock test tube, diluted twice with PBS, and incubated for 45 min with 150 μL of non-commercial monoclonal IgE anti-MAGE antibodies. Concurrently, the standard of serially diluted low-molecular mass MAGEs (LMW-MAGEs) was prepared with an antibody incubation step analogous to the plasma samples. A total of 100 μL of the antibody sample solution was then transferred to coated plates and incubated for two hours. The plates were washed three times with PBST. A solution of horseradish peroxidase-conjugated rabbit IgE (Acris Antibodies GmbH, Herford, Germany) diluted 1:7000 in PBS was added and the plates were incubated at room temperature for 2 h. After three washes with PBST, an o-phenylenediamine dihydrochloride (OPD, Sigma-Aldrich, Darmstadt, Germany) substrate was added and the plates were incubated at room temperature for 5 min. The absorbance was read at 450 nm with an Enspire plate reader (Perkin Elmer, Waltham, MA, USA). The AGE10 content of each sample was calculated using the standard curves. The LMW-MAGE, HMW-MAGE, and non-commercial anti-MAGE antibodies were prepared as described previously [24].

### 2.4. Statistical Analyses

The statistical analyses were performed using R v4.0.3 [26]. All results were represented as means with corresponding standard deviations (SD). The statistical significance was determined at a *p*-value under 0.05. As the Shapiro–Wilk test revealed a significant deviation of AGE10 concentrations from the normal distribution, we used non-parametric statistical tests. Group-wise comparisons were performed using the Mann–Whitney U-test. Correlations between the age, ALT, AST, GGTP, and bilirubin and the AGE10 concentration were assessed using Kendall′s rank correlation. To examine the diagnostic accuracy of AGE10, the sensitivity and specificity were calculated and a receiver operating characteristic (ROC) plot [27] was constructed. The optimal test cut-off value was determined using Youden′s J statistic [28], which was calculated with the following equation:

Higher *J* values corresponded with a higher test accuracy for a given cut-off value.

## 3. Results

### 3.1. Impact of the Demographic Characteristics of the Study Population on AGE10

Detailed characteristics of the study population are provided in Table 1. The plasma concentration of AGE10 did not differ between sexes (*p* = 0.54) or across ages (*p* = 0.29).

### 3.2. Impact of Alcohol Consumption and Liver Function on AGE10

**Figure 1:**
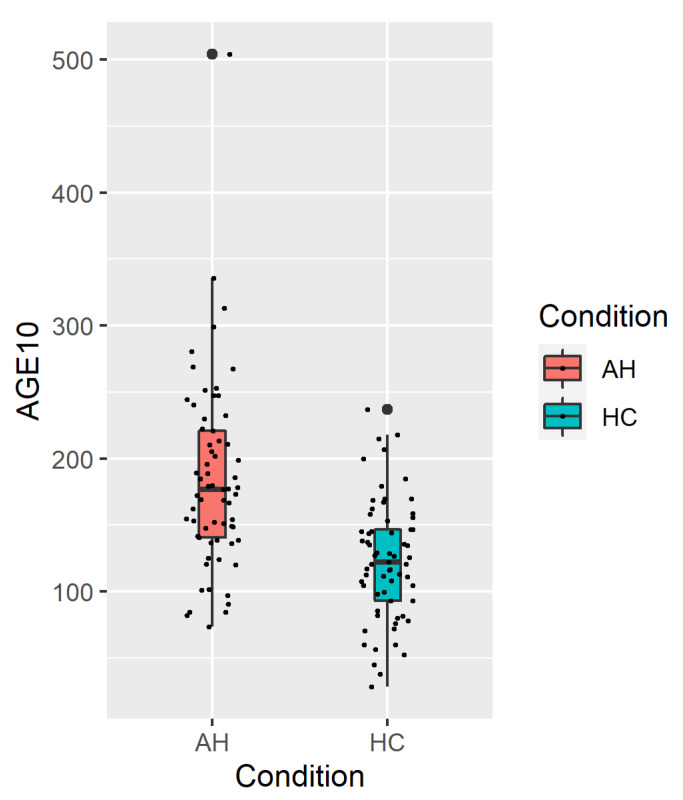
AGE10 concentration (µg/mL) in groups. AH: alcoholic hepatitis; HC: healthy control.

**Figure 2:**
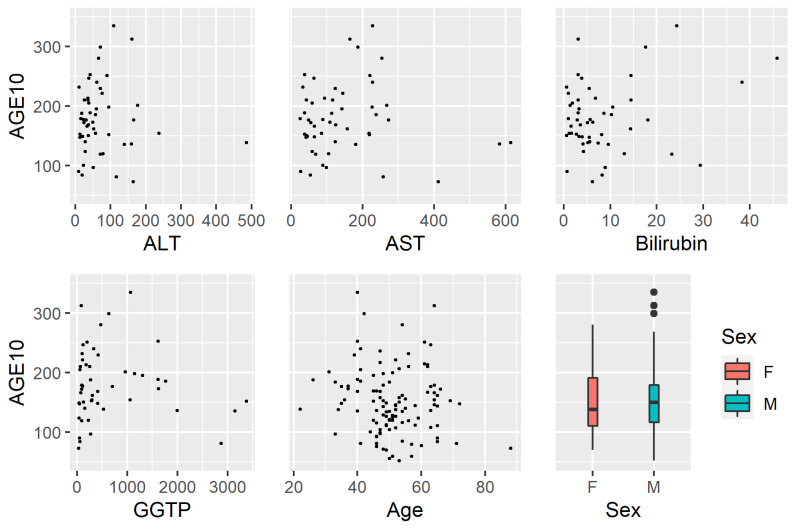
Association between AGE10 and patient characteristics.

The mean concentration of AGE10 was significantly higher in the AH group than in the healthy controls (184.5 ± 71.1 μg/mL and 123.5 ± 44.9 μg/mL, respectively; *p* < 0.001, Figure 1). There was no significant correlation between the AGE10 concentration and the markers of the liver function (AST, ALT, bilirubin, and GGTP with respective *p*-values of 0.53, 0.28, 0.86, and 0.30). The association between AGE10 and the study population characteristics is depicted in Figure 2.

### 3.3. Diagnostic Accuracy of AGE10 in Distinguishing AH from HC

**Figure 3:**
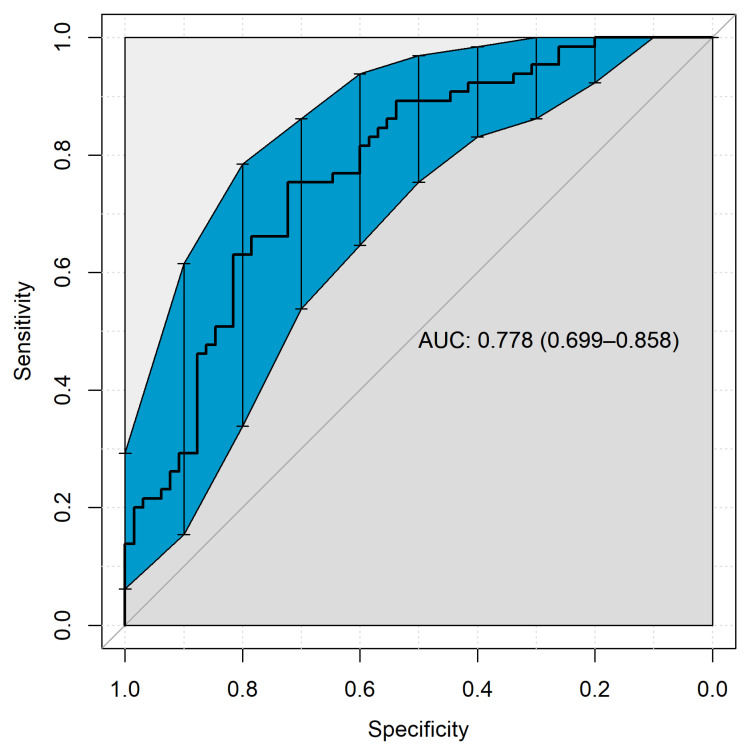
Receiver operating characteristic plot. The area in blue corresponds with 90% confidence intervals.

The diagnostic accuracy of AGE10 is presented as a ROC plot in Figure 3. The AGE10 concentration was able to distinguish AH from the healthy controls, with an AUC of 0.78; this is considered to be an acceptable performance for a diagnostic test [29]. The highest Youden′s *J* value was obtained at a cut-off value of 147.25 μg/mL, with a corresponding 75% sensitivity and 72% specificity.

## 4. Discussion

The objective of our study was to establish whether a novel subtype of AGEs, a melibiose-derived AGE analog, was associated with AH. We revealed that patients with AH possessed a higher plasma concentration of AGE10 than the healthy controls. In addition, we verified the diagnostic potential of AGE10 and confirmed that it had an acceptable performance in discriminating between AH and HC. Although the presence of AH was associated with significantly increased AGE10 levels, interestingly, there was no significant correlation between the AGE10 concentration and the ALT, AST, bilirubin, and GGTP levels.

Whether AGEs are a cause or a result of a liver injury remains a topic of ongoing discussion. The strongest support for AGEs as a result rather than a cause of a liver injury comes from Butscheid et al. They showed that the early stages of liver disease were not associated with significantly higher levels of CML or imidazolone compared with healthy controls [30]. Świderska et al. reported conflicting results; in their study, both advanced and early liver disease presented with significantly higher serum levels of AGEs than healthy controls (however, patients with advanced NAFLD had increased AGE levels compared with the early disease) [31]. Considering the existence of established mechanisms by which AGEs exert their harmful effect and the liver is involved in AGE clearance, it is most plausible that liver disease both causes and is caused, at least partially, by AGEs. The mechanism of a potential liver injury caused by AGEs is strongly intertwined with other pathophysiological events associated with ALD (i.e., microbiome alterations and ROS generation). There are two main mechanisms by which endogenous AGEs exert their harmful effects. Their direct action causes the impairment of proteins through cross-linking [32,33]. The other mechanism is an interaction between AGEs and their receptor (RAGE). The binding of AGEs to RAGE leads to the activation of multiple pathways associated with a liver injury, including nuclear factor-κB (NF-κB) and mitogen-activated protein kinase (MAPK)/c-Jun N-terminal protein kinase (JNK) pathways [32,33]. The involvement of NF-κB in liver injuries stems most likely from its proinflammatory effect [34]. The pathological activation of JNK leads to an increased production of ROS (which, through several downstream pathways, causes the subsequent and further activation of JNK, leading to a self-sustaining amplification loop) [35].

AGEs are provided to the body from both endogenous and exogenous sources. The major exogenous source is the consumption of a highly processed, Western diet and soft drinks high in fructose corn syrup [36]. The most important endogenous source of AGEs in patients abusing alcohol is alcoholysis in the liver, which results in the accumulation of acetaldehyde and the subsequent generation of acetaldehyde-derived AGEs [11]. However, the exact source of AGE10 remains a mystery. It remains to be elucidated whether MAGEs/AGE10 are present in the food and if they can be absorbed from the intestines. As for endogenous generation, the source of melibiose for MAGE/AGE10 generation is also not clear. There are two possible sources for supplying melibiose for an endogenous MAGE/AGE10 synthesis. Melibiose could be provided with food (such as honey [37] or a plant-based diet [38]) and then readily absorbed through paracellular junctions [39]. Another source for melibiose is fermentation by several bacterial genera present in the gut microbiome such as *Bifidobacterium*, *Lactobacillus*, or *Lactococcus* [40,41,42]. As AH is associated with increased relative abundances of *Lactobacillus* and *Bifidobacterium* [10], we considered microbial fermentation, and not supplementation by diet, to be the most plausible source of melibiose and, subsequently, a potential explanation for the increased AGE10 concentration in AH.

The role of exogenous (I.e., dietary) AGEs remains an important area of study. The absorption rate of AGEs depends on their chemical characteristics and varies between 10 and 30% [13]. The AGE content in food depends both on the characteristics of the raw product and on the way in which it was prepared. Generally, the AGE content is highest in animal-derived, fatty, and protein-rich foods. The amount of AGEs in a single, standard serving varies from values as low as 20 kU (in certain soy-derived products) to exceeding 10,000 kU (in highly processed meats) [43]. As up to 30% of dietary AGEs are absorbed, and as the AGE content in several foods is extremely high, the exogenous supply of AGEs is typically higher than the endogenous production. This led us to the conclusion that dietetic intervention is a valid approach for reducing plasma AGE levels. Although the impact of a low AGE diet on liver disease has not yet been evaluated in human subjects, there is indirect evidence supporting its protective effect. A recent meta-analysis confirmed that a low AGE diet resulted in a significant reduction of insulin resistance, fasting glucose, total cholesterol, and LDL [44]. In addition, a high AGE diet aggravated liver injuries in a murine model [45]. Several practical guidelines have already been proposed to reduce the intake of dietary AGEs. Uribarri et al. provided a convenient database describing the AGE (CML) content of 549 different types of food [43]. Apart from avoiding food with an inherently high AGE content, simple changes in food preparation might produce a staggering reduction in AGEs. Examples include marinating food in vinegar (which reduces the formation of AGEs through an increase in the pH), avoiding frying, and a reduction of the cooking time and temperature [46,47].

The pathophysiological impact of dietary AGEs is not limited to their contribution to the blood AGE pool. Dongen et al. showed that dietary AGEs induced changes in the mice microbiome [48]. Multiple studies have confirmed that ALD is associated with alterations in the gut microbiome [10]. The benefits of a fecal microbiota transplant (FMT) in ALD were confirmed in a recent placebo-controlled, double-blinded, randomized clinical trial. The FMT group had a significantly lower rate of AUD-related serious adverse effects compared with the placebo [49]. Interestingly, microbiome alterations associated with a high AGE diet and with AH showed an overlap. In both AH and a high AGE diet, melibiose-producing *Bifidobacterium* was enriched [48]. This result suggests the potential involvement of dietary AGEs in microbiome alterations associated with a liver injury. Due to fermentation-dependent melibiose production in the gut microbiome, we hypothesized that the relationship between AGEs and the microbiome might be bidirectional, with AGEs influencing the microbiome and the microbiome providing substrates for further AGE synthesis.

The biomarkers associated with a liver injury can be broadly divided into two groups: the direct markers of a hepatocyte injury and the markers of the immune response. Currently, the most promising non-invasive biomarkers of a liver injury are the M65 and M30 serum cytokeratin-18 epitopes. CK18 is an intermediate filament protein abundantly expressed in multiple cell types, including hepatocytes [50]. The M30 epitope is produced through caspase-mediated cleavage and the M65 epitope includes both caspase-cleaved and full-length CK18 [51]. Consequently, specifically targeting the M30 epitope is a specific marker of early apoptosis, and the M65 epitope is a more general marker of cell death [52]. Both M30 and M65 CK18 have been proposed as potential biomarkers of a liver injury. As hepatocyte death occurs in multiple types of liver disease, CK18 epitopes are not specific for an alcohol-induced injury. Mueller et al. [9] confirmed that although both M30 and M65 were associated with multiple histological parameters related to liver injuries, neither could differentiate patients with ALD from the NAFLD group. A recent, large meta-analysis incorporating 41 studies examining the diagnostic accuracy of M30 and M65 epitopes for the detection of non-alcoholic steatohepatitis and fibrosis revealed that the AUC significantly differed across the studies (from 0.69 to 0.82 for M30 and 0.69 to 0.91 for M65 [53]). Although the majority of the studies on CK18 were performed on patients with non-alcoholic liver disease, there are reports that the diagnostic performance for ALD is similar. With an AUC of 0.78, AGE10 is a promising biomarker for AH. This AUC puts the accuracy of AGE10 on a par with M30 and M65 (with respective AUCs of 0.776 and 0.784 for ALD [9]), the epitopes of cytokeratin-18. Apart from biomarkers that have been focused directly on detecting liver damage, there are multiple studies that have examined the accuracy of detecting factors related to the immune response associated with a liver injury, including soluble CD163 (sCD163), ST2 receptor, and several proinflammatory microRNAs (such as miRNA-192 [54]) [55]. CD163 is a macrophage- and monocyte-specific hemoglobin–haptoglobin scavenger receptor [56]. During inflammation, metalloproteinase cleaves CD163 near the cell membrane, resulting in the release of sCD163 into the circulation [57]. Increased levels of sCD163 have been detected in multiple types of liver injury, including HCV-induced [58], Wilson′s disease, and alcoholic hepatitis [59]. The diagnostic accuracy of sCD163 has been seldom reported and the studies mostly focused on examining its potential as a prognostic factor. In a study on acute liver disease with multiple etiologies, sCD163 was shown to predict a fatal outcome, with an AUC ranging from 0.64 to 0.8 [60]. ST2 is a receptor for interleukin-33 (IL-33) and exists in two forms, full-length and soluble (sST2) [61]. Similar to sCD162, sST2 has been studied mainly as a prognostic factor. Higher levels of sST2 are correlated with more severe stages of ALD [61]; however, to our knowledge, none of the studies on the topic have examined the diagnostic accuracy in discriminating the stages of ALD or predicting fatal outcomes. microRNA-192 (miRNA-192) is the second (after miRNA-122) most abundant microRNA expressed in the liver [62]. The expression of miRNA-192 was significantly larger in patients with alcoholic hepatitis compared with healthy controls [54]. In a recent study by Kim et al. [63], the authors were able to discriminate patients with a fatty liver from a group with steatohepatitis, with and AUC of 0.771. The performance was even better when using panels combining 4 or 8 microRNAs, with respective AUCs of 0.875 and 0.924. Although the preliminary results regarding the use of immunological markers for the diagnosis and prediction of ALD severity are promising, this approach has several shortcomings. The inflammatory response is present in multiple forms of liver injury (including NASH, ALD, or HBV-induced liver injury); hence, detecting higher levels of inflammation-associated biomarkers does not help to determine the underlying etiology. In addition, most of the inflammation-associated biomarkers are not liver-specific; for example, higher levels of sST2 have been detected in multiple highly inflammatory states other than liver disease such as aortic dissections, heart failure, and sepsis [64,65,66].

Similar to the M30 and M65 epitopes of CK18, the diagnostic use of AGEs in the context of liver disease has been mainly focused on NAFLD [17]. Panels that incorporated a soluble variant of the AGE receptor (sRAGE) along with different types of AGEs performed significantly better than only measuring AGEs (with an AUC ranging from below 0.78 up to 0.85) [67]. The performance of AGEs in differentiating a non-alcoholic fatty liver from non-alcoholic steatohepatitis was acceptable, with an AUC of 0.78 (which was associated with a relatively high specificity of 88.9% and a weak sensitivity of 66.7%) [68]. A similar pattern of a relatively high specificity (84%) and a low sensitivity (70%) was observed in the diagnostic accuracy of discriminating low-grade hepatic steatosis from moderate [31]. Although several authors have confirmed that ALD is associated with an increased concentration of classic subtypes of AGEs (such as CML and GA-AGE), none have provided the diagnostic accuracy for discriminating between ALD and healthy controls or between the different stages of ALD [69,70,71].

Our study had several limitations. First, the diagnosis was established by clinical criteria and not a biopsy. Although this approach is widely used in both scientific publications and in clinical practice, it is worth noting that a liver biopsy is the method with the highest sensitivity and specificity in the diagnosis and grading of ALD. The use of a biopsy would have allowed us to detect whether the AGE10 concentration significantly correlated with any individual histological features of ALD such as ballooning, necrosis, or fibrosis. However, due to the preliminary nature of our study, the use of an invasive diagnostic method associated with a relatively high risk of serious complications could not be justified. The majority of the AH group included in our study had a high Maddrey score, which further contributed to the increased risk of a biopsy. Although the alcoholic hepatitis group and healthy controls included in our study were age- and sex-matched, there were other potentially important confounding factors that could have influenced our results. As dietary AGEs are generally important contributors to the AGE pool (however, whether that is the case for AGE10 remains to be elucidated), we could not exclude the possibility that potential differences in the dietary habits between the groups also contributed to the different AGE10 concentrations. Another limitation was the lack of a comparison group with other liver disorders. Based on the data from our study, it was impossible to tell whether the AGE10 increase was specific to alcoholic liver disease or if it would be elevated in other types of liver injury such as NAFLD or viral infections. Determining these associations is an important direction for our subsequent studies. In conclusion, our study showed that AH was associated with significantly increased plasma AGE10 levels and that the AGE10 concentration exhibited an acceptable diagnostic accuracy in distinguishing between patients with AH and healthy controls.

## Figures and Tables

**Table 1:** Characteristics of the study population.

Parameters	HC	AH
Age	52	49.62
Sex (M/F)	44/21	45/20
AST	-	144.67
ALT	-	71.45
Bilirubin	-	8.46
GGTP	-	638.65

## Data Availability

Data supporting the results will be made available by the authors upon reasonable request.
